# Data of the Modified Somatic Perception Questionnaire (MSPQ) administered to a sample of immigrants in Genoa (Italy)

**DOI:** 10.1016/j.dib.2017.04.048

**Published:** 2017-05-04

**Authors:** Nicola Luigi Bragazzi, Hicham Khabbache, Ali Assad Watfa, Anna Siri, Mariano Martini, Diana Spulber, Tania Simona Re, Werner Maria Natta, Giovanni Del Puente

**Affiliations:** a School of Public Health, Department of Health Sciences (DISSAL), University of Genoa, Genoa, Italy; b UNESCO CHAIR “Anthropology of Health – Biosphere and Healing System”, University of Genoa, Genoa, Italy; c Department of Neuroscience, Rehabilitation, Ophthalmology, Genetics, Maternal and Child Health, Section of Psychiatry, University of Genoa, Genoa, Italy; d Laboratoire Etudes théologiques, Sciences Cognitives et Sociales, Faculty of Literature and Humanistic Studies, Sais, Sidi Mohamed Ben Abdellah University, Fez, Morocco; e Faculty of Education, Kuwait University, Kuwait City, Kuwait

**Keywords:** Immigration, Modified Somatic Perception Questionnaire (MSPQ), Psychometric properties, Somatization

## Abstract

This article reports the data of the Modified Somatic Perception Questionnaire (MSPQ) administered to a sample of 143 immigrants accessing an outpatient clinic or the general practitioners offices in Genoa (Italy) compared with 186 Italian patients. For further details and for the interpretation of the data, the reader is referred to the original publication “Somatic perception, cultural differences and immigration: results from administration of the Modified Somatic Perception Questionnaire (MSPQ) to a sample of immigrants” by Bragazzi et al. (2014) [1].

**Specifications Table**

**Table:** 

Subject area	*Psychology*
More specific subject area	*Immigration psychology*
Type of data	*Table, graphs*
How data was acquired	*Administration of a questionnaire in its Italian version to a sample of immigrants*
Data format	*Raw, analyzed*
Experimental factors	*Questionnaire scores*
Experimental features	*Psychometric properties, including Cronbach׳s alpha coefficient, and partial least-square structural equation modeling of the questionnaire in its Italian version administered to a sample of immigrants*
Data source location	*Genoa, Italy*
Data accessibility	*Data are within this article*

**Value of the data**

• Scores and psychometric properties of the Modified Somatic Perception Questionnaire (MSPQ) administered to a sample of immigrants in Genoa (Italy) are reported.
• These data could be useful for the scientific community in providing insights about the usefulness of the MSPQ, especially when working with immigrant subjects.
• These data could be further replicated in order to demonstrate the usefulness and validity of the MSPQ in a more robust way.

## 1 Data

**Fig. 1:**
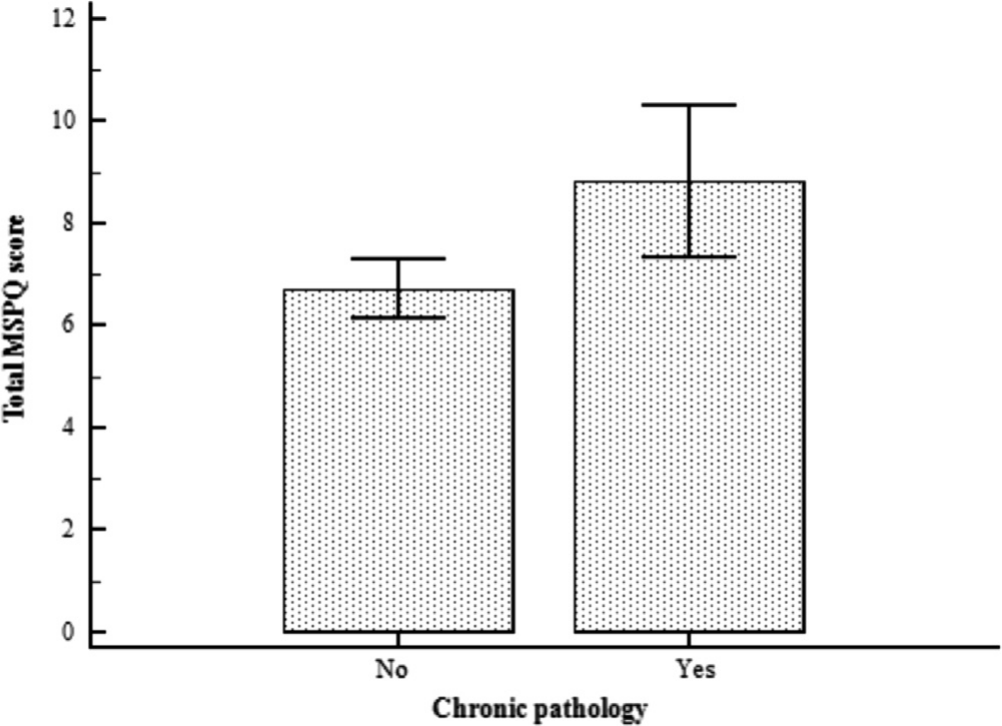
Independent sample Student׳s *t*-test investigating the impact of the presence of a chronic pathology on the total score of the Modified Somatic Perception Questionnaire (MSPQ).

**Fig. 2:**
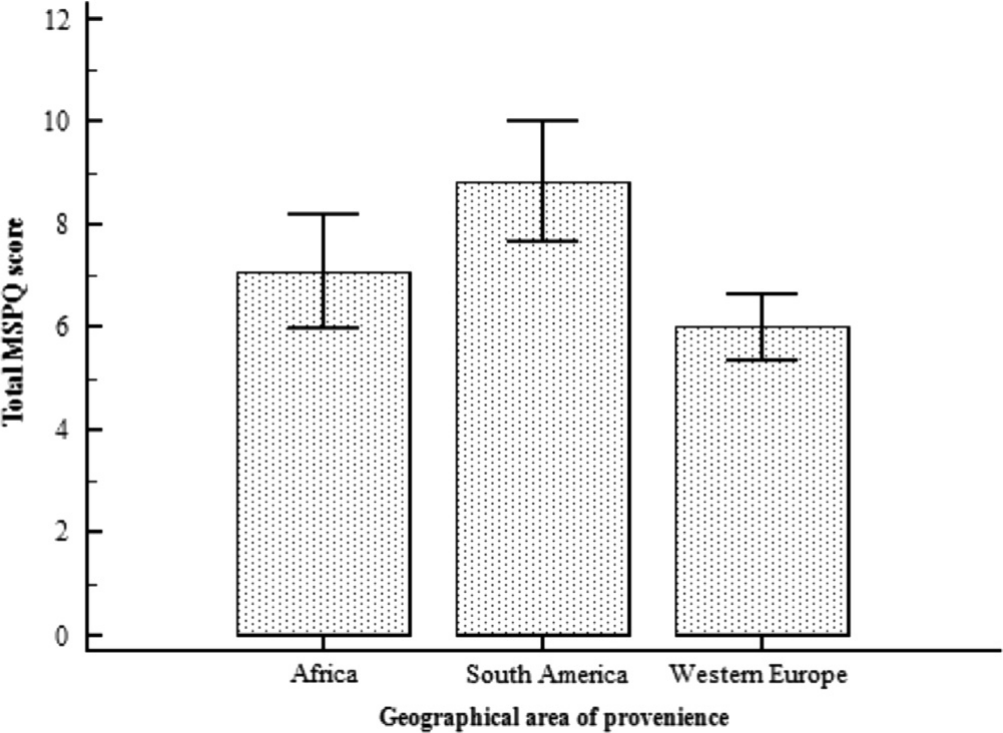
1-way ANOVA investigating the impact of the geographic provenience on the total score of the Modified Somatic Perception Questionnaire (MSPQ).

**Fig. 3:**
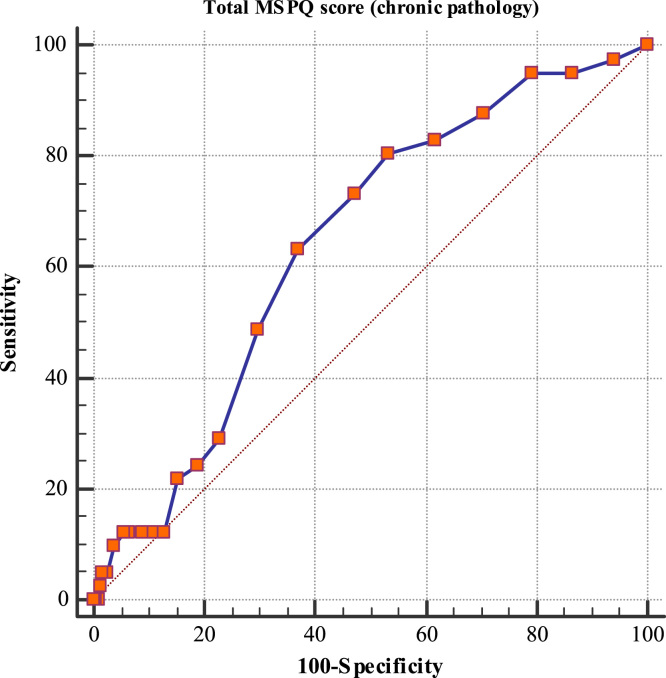
Receiver operating characteristic (ROC) analysis investigating the impact of the presence of a chronic pathology on the total score of the Modified Somatic Perception Questionnaire (MSPQ).

**Fig. 4:**
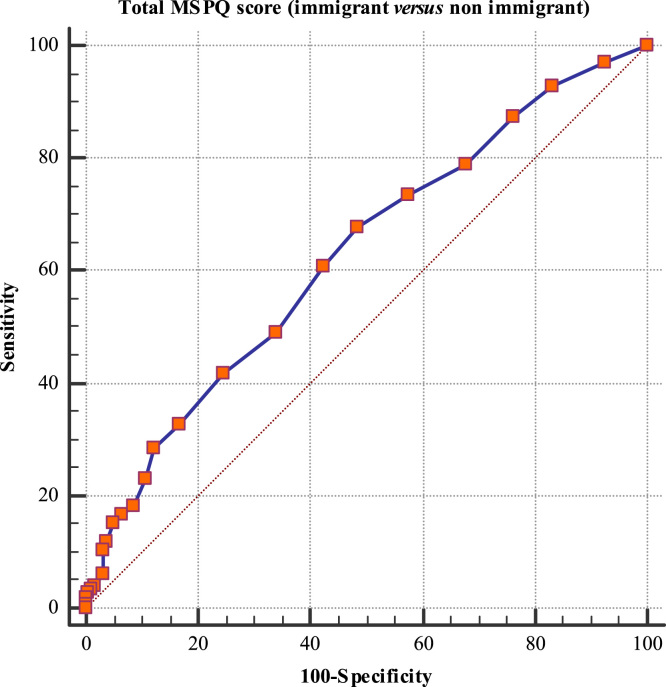
Receiver operating characteristic (ROC) analysis investigating the impact of the geographic provenience (immigrant *versus* non immigrant) on the total score of the Modified Somatic Perception Questionnaire (MSPQ).

**Fig. 5:**
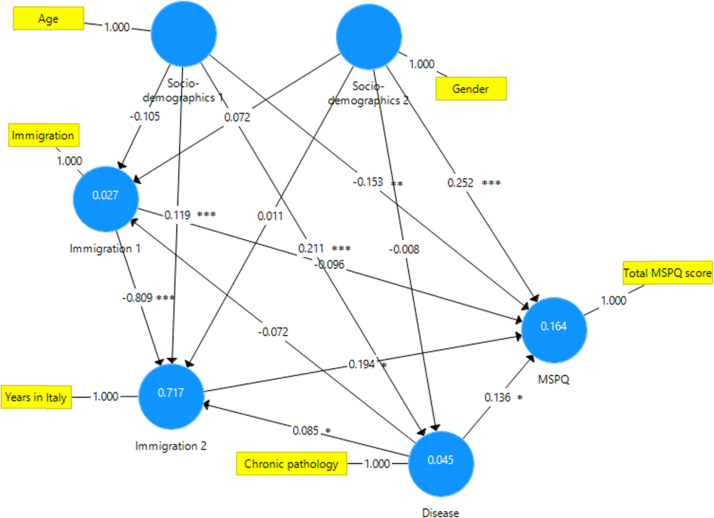
Structural equation model investigating the relationship between the variables and the total Modified Somatic Perception Questionnaire (MSPQ) score. *Statistically significant with *p*-value<0.05; **Statistically significant with *p*-value<0.01; ***Statistically significant with *p*-value<0.001.

**Fig. 6:**
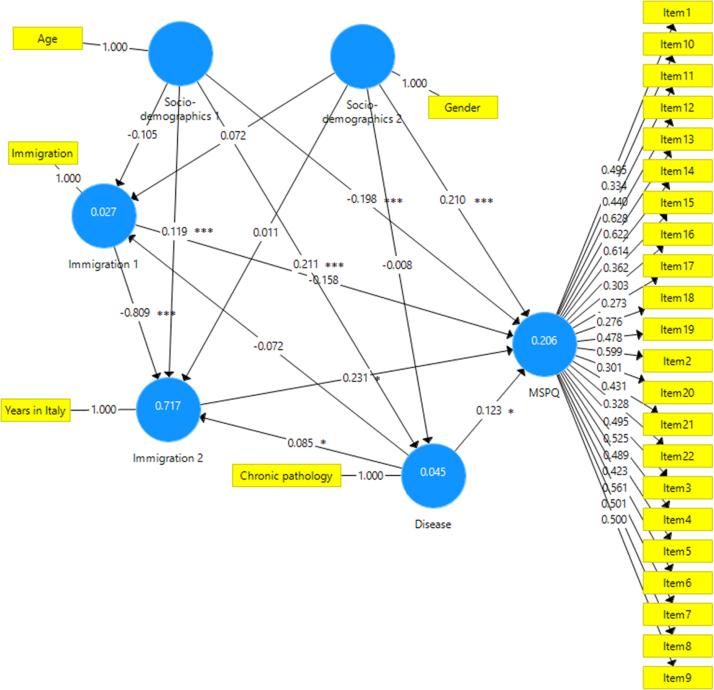
Structural equation model investigating the relationship between the variables and the total Modified Somatic Perception Questionnaire (MSPQ) scores for each item. *Statistically significant with *p*-value<0.05; **Statistically significant with *p*-value<0.01; ***Statistically significant with *p*-value<0.001.

This paper contains the scores and the psychometric properties of the Modified Somatic Perception Questionnaire (MSPQ) administered in its Italian version to a sample of 143 immigrants in Genoa (Italy) compared with a sample of 186 Italian patients. Univariate and multivariate descriptive statistics is reported in Table 1, Table 2 and pictorially shown in Figs. 1 and 2, with the fit indexes of the 1-factor model reported in Table 3, whilst results of the receiver operator characteristic (ROC) analysis are shown in Table 4, Table 5, Table 6, Table 7, 6 and 7, and pictorially shown in Fig. 3, Fig. 4, the outcomes of the 2-way analysis of variance (ANOVA) analysis are tabulated in Table 8, whilst Table 9 reports the overall Cronbach׳s alpha coefficient and the effect of dropping each item. Structural models are shown in Fig. 5, Fig. 6. For further details (such as the composition of the sample) and the interpretation and discussion of the data, the reader is referred to the original publication [1].

**Table 1:** Scores (mean and standard deviation) and descriptive statistics (skewness and kurtosis) for each item of the Modified Somatic Perception Questionnaire (MSPQ).

**Items**	**Mean**	**Standard deviation**	**Skewness**		**Kurtosis**	
			**Statistics**	**Standard error**	**Statistics**	**Standard error**
Heart rate increase	0.73	0.777	0.685	0.159	−0.487	0.317
Feeling hot all over	0.86	0.930	0.602	0.134	−0.897	0.268
Sweating all over	0.68	0.883	1.179	0.134	0.528	0.268
Sweating in a particular part of the body	0.71	0.803	0.825	0.159	−0.211	0.317
Pulse in neck	0.34	0.610	1.828	0.159	3.172	0.317
Pounding in head	0.35	0.632	1.834	0.159	2.909	0.317
Dizziness	0.40	0.607	1.343	0.134	1.133	0.268
Blurred vision	0.38	0.610	1.591	0.135	2.536	0.268
Feeling faint	0.33	0.601	1.999	0.134	4.284	0.268
Everything appearing unreal	0.17	0.526	3.557	0.159	13.069	0.317
Nausea	0.36	0.590	1.478	0.134	1.593	0.268
Butterflies in stomach	0.79	0.839	0.754	0.159	−0.265	0.317
Pain or ache in stomach	0.71	0.816	0.828	0.134	−0.314	0.268
Stomach churning	0.67	0.794	0.989	0.134	0.293	0.268
Desire to pass water	0.53	0.753	1.311	0.159	1.077	0.317
Mouth becoming dry	0.57	0.738	1.030	0.134	0.081	0.268
Difficulty swallowing	0.20	0.486	2.714	0.159	7.920	0.317
Muscles in neck aching	0.60	0.839	1.265	0.134	0.727	0.268
Legs feeling weak	0.75	0.814	0.930	0.134	0.324	0.268
Muscles twitching or jumping	0.43	0.659	1.454	0.134	1.607	0.268
Tense feeling across forehead	0.25	0.583	2.497	0.134	5.995	0.268
Tense feeling in jaw muscles	0.17	0.459	3.020	0.159	10.112	0.317

**Table 2:** Analysis of the Mardia׳s multivariate asymmetry skewness and kurtosis.

**Parameter**	**Coefficient**	**Statistic**	**Degrees of freedom**
Skewness	174.812	10,051.689	2024
Corrected skewness	174.812	10,146.745	2024
Kurtosis	804.681	79.073***	

*Statistically significant with *p*-value<0.05; **Statistically significant with *p*-value<0.01.

*** Statistically significant with *p*-value<0.001.

**Table 3:** Fit indexes for the 1-factor model of the Modified Somatic Perception Questionnaire (MSPQ), performing a parallel analysis (PA) on a polychoric correlations-based dispersion matrix. Abbreviations: AGFI (Adjusted Goodness of Fit Index); BIC (Schwarz׳s Bayesian Information Criterion); CFI (Comparative Fit Index); GFI (Goodness of Fit Index); NNFI (Non-Normed Fit Index or Tucker & Lewis Index); RMSEA (Root Mean Square Error of Approximation).

**Fit index**	**Value**	**Bootstrap 95% CI**
Minimum Fit Function Chi Square with 209 degrees of freedom	931.405 (*p*=0.000010)	
Robust Mean-Scaled Chi Square with 209 degrees of freedom	758.641 (*p*=0.000010)	
Chi-Square for independence model with 231 degrees of freedom	9114.742	
RMSEA	0.086	0.082–0.087
NNFI	0.932	0.923–0.948
CFI	0.938	0.930–0.953
BIC	993.421	945.155–1015.757
GFI	0.928	0.923–0.941
AGFI	0.920	0.915–0.934

**Table 4:** Receiver operating characteristic (ROC) analysis when discriminating immigrant *versus* non immigrant groups.

**Area under the ROC curve (AUC)**	
Area under the ROC curve (AUC)	0.627±0.0309
95% Confidence interval	0.572–0.679
z statistic	4.105
Significance level P (Area=0.5)	<0.0001***

**Youden index**	
Youden index J	0.1945
Sensitivity	67.83
Specificity	51.61

*Statistically significant with *p*-value<0.05; **Statistically significant with *p*-value<0.01.

*** Statistically significant with *p*-value<0.001.

**Table 5:** Sensitivity and specificity values and area under the curve (AUC) for each item of the Modified Somatic Perception Questionnaire (MSPQ) in distinguishing between an immigrant and an Italian subject.

**Item**	**Sensitivity**	**Specificity**	**AUC**	***p*-value**
Item 1	51.3	59.3	0 .535	0.3067
Item 2	42.4	83.9	0 .656	<0.0001***
Item 3	54.4	62.9	0 .596	0.0005***
Item 4	55.7	59.3	0 .571	0.0383*
Item 5	5.1	93.4	0 .503	0.9197
Item 6	31.0	76.9	0 .539	0.1832
Item 7	49.4	78.0	0.642	<0.0001***
Item 8	37.6	72.0	0.550	0.0525
Item 9	31.0	75.8	0.537	0.1314
Item 10	91.1	15.4	0.533	0.1369
Item 11	96.2	6.5	0.510	0.6844
Item 12	1.3	93.4	0.522	0.5408
Item 13	24.1	86.0	0.562	0.0290*
Item 14	59.5	60.2	0.600	0.0003***
Item 15	69.0	51,6	0.613	0.0007***
Item 16	9.5	83.9	0.513	0.6453
Item 17	86.7	22.0	0.543	0.0975
Item 18	88.0	17.2	0.529	0.2950
Item 19	58.9	49.5	0.548	0.0980
Item 20	38.6	69.9	0.530	0.2513
Item 21	24.1	86.0	0.550	0.0189*
Item 22	88.6	18.7	0.535	0.1435

**Statistically significant with *p*-value<0.01.

* Statistically significant with *p*-value<0.05.

*** Statistically significant with *p*-value<0.001.

**Table 6:** Receiver operating characteristic (ROC) analysis when discriminating for the presence of a chronic pathology.

**Area under the ROC curve (AUC)**	
Area under the ROC curve (AUC)	0.643±0.0419
95% Confidence interval	0.589–0.695
z statistic	3.419
Significance level P (Area=0.5)	0.0006***

**Youden index**	
Youden index J	0.2702
Sensitivity	80.49
Specificity	46.53

*Statistically significant with *p*-value<0.05; **Statistically significant with *p*-value<0.01.

*** Statistically significant with *p*-value<0.001.

## 2 Experimental design, materials and methods

Descriptive statistics analyses (mean and standard deviation, skewness and kurtosis) were carried out. Independent sample Student׳s test, analysis of variance/ANOVA one- and two-ways, ROC analyses were performed in order to assess the impact of geographic provenience and presence of a chronic pathology on the total MSPQ score. Figures with *p*-value<0.05 were considered statistically significant. Statistical analyses were performed using the commercial software Statistical Package for Social Sciences (SPSS v22.0; IBM Corporation, Armonk, NY, USA). The factorial structure was assessed using the software Factor Analysis for Windows (v10.4.01, Rovira i Virgili University, Tarragona, Spain), performing a Parallel Analysis (PA) on a polychoric correlations–based dispersion matrix. A partial least-square structural equation model was carried out using the SmartPLS software v3.2.6 for Windows.

**Table 7:** Sensitivity and specificity values and area under the curve (AUC) for each item of the Modified Somatic Perception Questionnaire (MSPQ) in distinguishing between a subject with and without a chronic pathology.

**Item**	**Sensitivity**	**Specificity**	**AUC**	***p*-value**
Item 1	66.7	49.8	0.590	0.0607
Item 2	60.9	49.3	0.552	0.2222
Item 3	6.5	82.9	0.511	0.7788
Item 4	94.4	18.3	0.559	0.1709
Item 5	11.1	95.3	0.537	0.4010
Item 6	36.1	73.2	0.549	0.2659
Item 7	43.5	66.8	0.549	0.2181
Item 8	39.1	68.7	0.547	0.2463
Item 9	8.7	96.0	0.523	0.5451
Item 10	2.8	98.6	0.500	0.9946
Item 11	43.5	70.8	0.570	0.0765
Item 12	66.7	46.5	0.579	0.1077
Item 13	69.6	54.0	0.637	0.0011**
Item 14	67.4	54.0	0.618	0.0046**
Item 15	5.6	98,1	0.504	0.9390
Item 16	54.3	59.7	0.572	0.0817
Item 17	88.9	17.4	0.531	0.3086
Item 18	89.1	15.4	0.505	0.8915
Item 19	67.4	47.7	0.581	0.0513
Item 20	52.2	68.8	0.590	0.0200*
Item 21	21.7	81.9	0.515	0.6358
Item 22	88.9	14.6	0.517	0.5598

***Statistically significant with *p*-value<0.001.

* Statistically significant with *p*-value<0.05.

** Statistically significant with *p*-value<0.01.

**Table 8:** 2-way ANOVA assessing the impact of immigration, presence of a chronic pathology and interaction immigration X chronic pathology on the total score of the Modified Somatic Perception Questionnaire (MSPQ).

**Source**	**F**	**Degrees of freedom**	**P**
Immigration	0.725	1	0.395
Chronic pathology	6.021	1	0.015*
Immigration×Chronic pathology	6.108	1	0.014*

**Statistically significant with *p*-value<0.01; ***Statistically significant with *p*-value<0.001.

* Statistically significant with *p*-value<0.05.

**Table 9:** Overall Cronbach׳s alpha coefficient and the effect of dropping each item.

**Item**	**Cronbach׳s alpha**	**Change**
Item 1	0.8279	−0.002816
Item 2	0.8217	−0.009051
Item 3	0.8259	−0.004822
Item 4	0.8241	−0.006608
Item 5	0.8252	−0.005517
Item 6	0.8264	−0.004353
Item 7	0.8213	−0.009438
Item 8	0.8223	−0.008492
Item 9	0.8212	−0.009584
Item 10	0.8260	−0.004745
Item 11	0.8248	−0.005907
Item 12	0.8223	−0.008431
Item 13	0.8199	−0.01084
Item 14	0.8188	−0.01197
Item 15	0.8276	−0.003128
Item 16	0.8269	−0.003879
Item 17	0.8263	−0.004469
Item 18	0.8260	−0.004744
Item 19	0.8220	−0.008740
Item 20	0.8258	−0.004932
Item 21	0.8214	−0.009363
Item 22	0.8256	−0.005164
Overall	0.8308	
